# Editorial: Environmental or occupational exposure to optical radiation: risk evaluation, health effects and prevention - tangible innovation for public and occupational health? volume II

**DOI:** 10.3389/fpubh.2026.1812676

**Published:** 2026-03-25

**Authors:** Alberto Modenese

**Affiliations:** Department of Biomedical, Metabolic and Neural Sciences, University of Modena and Reggio Emilia, Modena, Italy

**Keywords:** eye protection, occupational exposure, optical radiation, skin cancer, welding

Optical radiation (OR), encompassing wavelengths from ultraviolet (UV) to infrared (IR), represents a pervasive environmental and occupational exposure that has profound implications for human health. Excessive or poorly managed exposure, especially to solar UV radiation, substantially contributes to adverse health outcomes such as skin cancers and ocular damage and remains a leading concern for public health, occupational safety, and preventive medicine (1, 2).

This second volume of the Research Topic on *Environmental or occupational exposure to optical radiation: risk evaluation, health effects and prevention—Tangible innovation for public and occupational health?* seeks to advance scientific knowledge, methodological innovation, and translational impact for researchers, practitioners, and policymakers worldwide. A central aim is to highlight methodological progress in exposure assessment (3) and to frame actionable insights into disease burden, preventive strategies, and health surveillance systems in a changing, post-pandemic, climate-altered world (4). This Research Topic includes four high-impact contributions that together illustrate the breadth of current research challenges and opportunities in optical radiation exposure.

In the first original research article, Paulo et al. quantify the increased risk of squamous cell carcinoma (SCC) among outdoor workers in Lisbon based on real-world solar UV radiation measurements. Using personal UV dosimetry and occupational categorization, the authors demonstrate substantially elevated excess risks of SCC across multiple outdoor occupations, underscoring that chronic solar UV exposure remains an urgent occupational carcinogenic hazard. This study bridges exposure assessment with epidemiological risk quantification and highlights the need for improved occupational surveillance and prevention policies.

Complementing this epidemiological perspective, Aktas et al. propose a Human Sentinel Surveillance Platform designed to overcome the limitations of conventional exposure monitoring. Their perspective article outlines a scalable digital infrastructure that integrates biomarker-based monitoring, standardized questionnaires, and harmonized data systems to capture dynamic occupational and environmental exposures in real time. By emphasizing multidisciplinary data integration and adaptive surveillance frameworks, this work reflects a forward-looking approach to exposome research and population health monitoring that can support early warning systems and evidence-based interventions.

The third contribution—a systematic review and meta-analysis by Atalay et al.—focuses on ocular protection practices among welders in sub-Saharan Africa. Welders experience intense OR from artificial sources, and this article synthesizes the prevalence, patterns, and determinants of protective behaviors. The article identifies critical gaps in the adoption of effective eye protection and emphasizes the interplay between occupational practices, resource constraints, and health education. The findings point to the importance of context-specific interventions that bridge occupational safety standards with on-the-ground realities in low-resource settings.

Rounding out the Research Topic, the fourth article by Sun et al. investigates the relationship between exposure to outdoor artificial light at night and the risk of gestational diabetes mellitus, extending the concept of optical radiation beyond UV to examine non-solar, artificial exposures and broader public health outcomes. This case-control study provides evidence that environmental light pollution, which is a growing concern in urbanized settings, may influence metabolic health during pregnancy, thus broadening the scope of optical radiation research to include circadian and non-visual health pathways.

Taken together, these contributions illustrate a continuum of research, from quantifying cancer risk and improving occupational exposure assessment to enhancing surveillance infrastructure, understanding protective behaviors, and exploring novel health endpoints associated with OR. They highlight the necessity of interdisciplinary approaches that unite exposure science, epidemiology, occupational health, and digital health innovations. Continued collaboration across these domains is crucial for developing evidence-based guidelines, effective prevention strategies, and policies that protect workers and the general public from the diverse health impacts of OR.
